# Association between lipid accumulation product and the risk of colon cancer in adults: A population-based study

**DOI:** 10.1371/journal.pone.0317462

**Published:** 2025-01-27

**Authors:** Renjie Guo, Weiming Wei

**Affiliations:** Department of Gastrointestinal Surgery, Fujian Provincial Hospital, Fuzhou, China; Tehran University of Medical Sciences, ISLAMIC REPUBLIC OF IRAN

## Abstract

**Background:**

The purpose of this study was to look into any potential connections between the occurrence of colon cancer and the condition of the body of lipid accumulation product (LAP) index.

**Methods:**

Using data from the 2009–2018 National Health and Nutrition Examination Survey (NHANES), we performed a cross-sectional analysis with 24,592 individuals. Utilizing multivariate logistic regression modelling, the relationship between LAP levels and colon cancer risk was investigated. Subgroup analysis, trend test, interaction test, and stratified smoothed curve were also carried out.

**Results:**

LAP levels and colon cancer risk were positively correlated after controlling for potential covariates (OR = 10.56, 95% CI: 2.40–46.53), the findings of trend tests are statistically significant. In particular groups, subgroup analysis revealed a positive connection between LAP levels and the risk of colon cancer. The association between LAP levels and colon cancer risk was shown to be M-shaped in the group under 60 years old, inverted V-shaped in the female and no-diabetes groups, and inverted L-shaped in the smoking and no-hypertensive groups, according to stratified smoothed curve fitting.

**Conclusions:**

According to our findings, there is a strong correlation between LAP levels and the risk of colon cancer.

## Introduction

One of the most prevalent malignant tumors of the gastrointestinal system, colon cancer, also called colorectal cancer (CRC), starts in the colon or rectum [1]. As the second largest cause of death from cancer around the world, colon cancer is one of the most prevalent malignancies [2]. The number of colon cancer cases and fatalities is increasing each year, according to statistics reports from the American Cancer Society [3]. These increases are endangering public health. The etiology and course of colon cancer are complex, with obesity and metabolic syndrome variables being major contributors [4, 5]. According to earlier research, white fat, as well as the inflammatory elements it is linked to, are important in the obesity-cancer relationship [6–8]. However, given that visceral fat represents a distinct subtype of white adipose tissue, further investigation is warranted to elucidate the specific mechanisms by which visceral adiposity contributes to the pathogenesis of colorectal cancer.

To evaluate lipid accumulation and metabolism in the body, the Lipid Accumulation Product (LAP), a recently suggested innovative index, integrates two important biological measures: triglycerides and waist circumference [9, 10]. According to some research, there is a direct link between obesity and colon cancer, and having a high body mass index (BMI) increases the risk of the disease [11–13]. Through inflammatory reactions and hormone production, obesity can also result in abnormalities in lipid metabolism in the body, such as higher blood triglyceride levels and insulin resistance, which may further contribute to the development of colon cancer [14, 15]. But in addition to reflecting obesity, LAP may also thoroughly indicate the degree of lipid metabolism issues by combining waist circumference and triglyceride level [16–18]. Consequently, it is imperative to aggressively investigate the connection between LAP and colon cancer, since this will be crucial for the practical implementation of novel blood indicators for colon cancer surveillance and prognostic evaluation.

This study assessed the possible relationship between LAP levels and colon cancer in Americans, a condition for which LAP is an emerging biomarker with significant promise. We took into account any relevant confounders and used survey data from a sizable population. Our research aims to add to the amount of information already available about the health impacts of LAP which might have some ramifications for early detection and colon cancer prevention methods.

## Methods

### Study design and population

**Fig 1:**
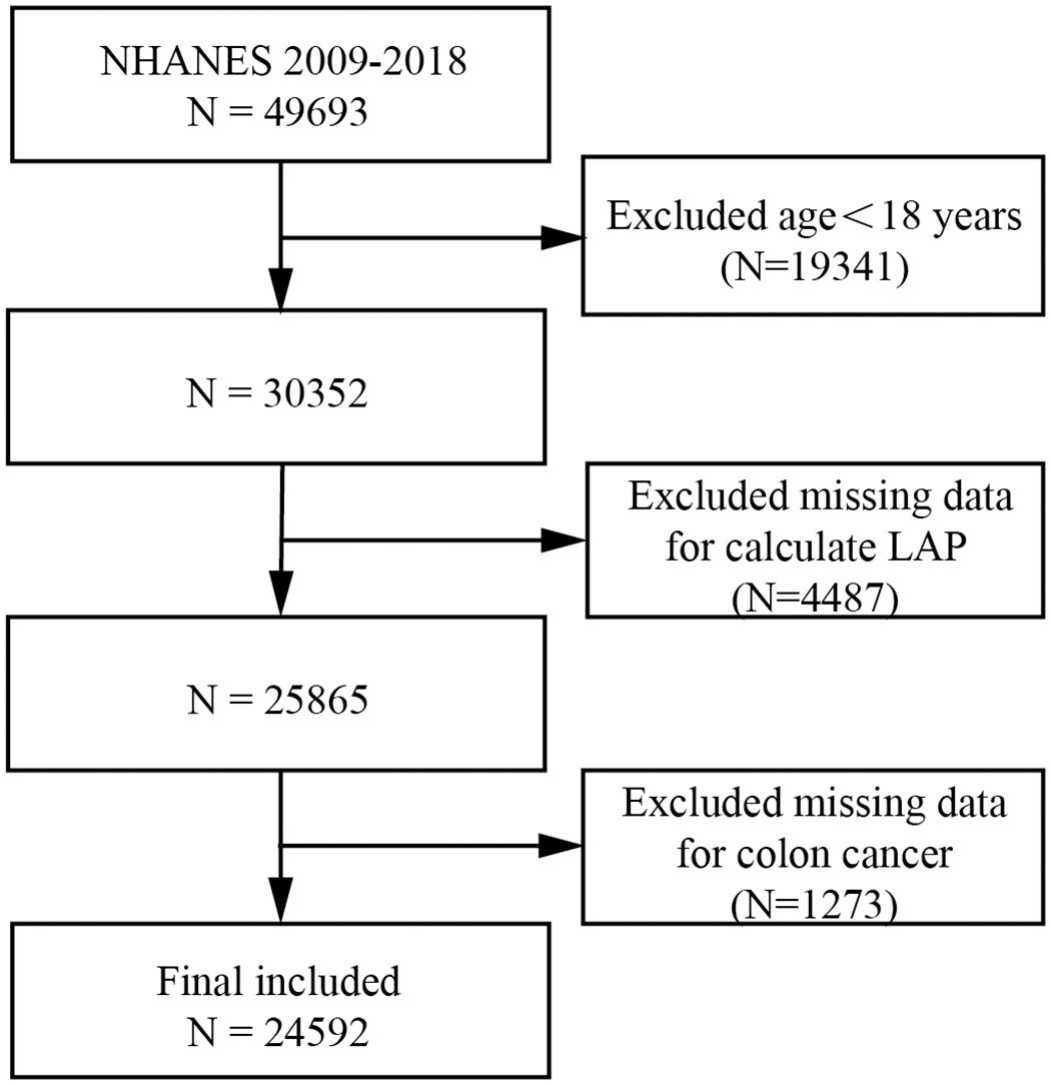
Flow chart.

The Centers for Disease Control and Prevention administer the NHANES, a comprehensive cross-sectional study with a nationwide representative sample. The National Center for Health Statistics Ethics Review Board authorized the study design, data collection, and survey techniques, and all participants gave their appropriate, written informed permission [19]. In the current work, we used five consecutive cycles of 10 years of NHANES data from 2009 to 2018, ensuring an appropriate sample size and trends over time for pertinent analysis. 49,693 of them were initially included in our study; however, to reduce possible bias in the analysis, we excluded individuals who were less than 18 years old, as well as missing data on colon cancer, WC, TG, and TG values more than 15 mmol/L. In the conclusion of our research population analysis, 24,592 people were included, as Fig 1.

### Variables

The level of LAP was the independent variable in this study, while colon cancer was the dependent variable. “Have you ever been told by a doctor or health professional that you have colon cancer?” and “Have you ever been told by a doctor or health professional that you have rectum cancer?” were the questions that participants were asked through a trained interviewer. It was suggested that there was a history of colon cancer if the response to any of these questions was “Yes”. We only regarded individuals as having a history of colon cancer if they had received a positive diagnosis of either colon or rectal cancer to reduce the possibility of misinterpretation bias.

At mobile screening facilities, highly skilled health specialists evaluate anthropometric measures as part of the NHANES. Specifically, a tape measure was used to measure the width of the superior edge of the iliac crest in centimeters (cm). For males and women, respectively, the LAP index was computed as [WC (cm) − 65] * [TG (mmol/L)] and [WC (cm) − 58] * [TG (mmol/L)] [20]. The initial procedure eliminated all subjects with blood TG values of more than 15 mmol/L, as well as men and women with chest circumferences of 65 cm and 58 cm, respectively. Measurements and computations, together with comprehensive details on these variables, are provided at www.cdc.gov/nchs/nhanes/.

### Statistical analysis

In contrast, the current study used sophisticated multistage cluster surveys and suitable sample weights to reduce dataset volatility while conducting a statistical analysis in compliance with pertinent centers for Disease Control and Prevention criteria. We utilized proportions to represent categorical variables and means±standard error to represent continuous variables [21, 22]. The existence or absence of colon cancer was then used to statistically assess descriptive analyses of the research population. Differences between groups were then ascertained using t-tests for continuous variables and chi-square testing for categorical variables. We utilized several linear regression models to account for relevant variables and examine the connection between LAP levels and the prevalence of colon cancer. The study employed three distinct models: the unadjusted model 1, model 2 was adjusted for age, gender, and race, and the fully adjusted model 3, which additionally included adjustments for age, gender, race, marital status, education level, ratio of family income to poverty, smoking, alcohol use status, hypertension, diabetes, TC, HDL, LDL, and BMI. The LAP was functionally converted (ln transform) in the regression analysis due to its skewness. The quartiles of LAP levels were examined after the continuous variable analysis. Odds ratios (ORs) and matching 95% confidence interval ratios (CIs) were used to represent the results. Corresponding trend analyses and sensitivity analyses were performed to investigate any possible trends. Furthermore, we investigated the nonlinearity of the relationship between LAP level and colon cancer risk in various population subgroups by hierarchical smoothed curve fitting. We also used interaction effects to test the heterogeneity of the association among subgroups and subgroup analyses to further explore the population specificity of the association between LAP level and colon cancer risk. ***P*** < 0.05 was regarded as statistically significant in all statistical analyses, which were conducted using Empower software (www.empowerstats.com) and R version 4.2.2 (http://www.R-project.org).

## Results

### Baseline characteristics of participants

This study had 24,592 individuals in total, of which 32.58% were 60 years of age or older and 67.42% were under that age. Conversely, Table 1 presents the pertinent baseline characteristics of the subjects as a column-stratified variable depending on whether or not they had colon cancer. Age, race, smoking, hypertension, diabetes, BMI, alcohol use status, and LAP were statistically significantly linked with the prevalence of colon cancer (***P*** < 0.05). In contrast to individuals without colon cancer, those with colon cancer typically had higher LAP levels, were older, non-Hispanic White, smoking, hypertension, and diabetes, had higher BMI, and had less alcohol use status.

**Table 1:** Characteristics of the study population based on colon cancer.

	Colon cancer (N = 148)	Non-colon cancer (N = 24444)	*P*-value
Age (years)	68.99±12.46	49.11±17.45	<0.001
Gender (%)			0.733
Male	47.30%	48.70%	
Female	52.70%	51.30%	
Race (%)			<0.001
Mexican American	7.43%	14.96%	
Other Hispanic	6.75%	10.55%	
Non-Hispanic White	62.84%	39.79%	
Non-Hispanic Black	16.22%	20.78%	
Other Races	6.76%	13.92%	
Marital status (%)			0.067
Married/Living with partner	52.38%	59.80%	
Widowed/Divorced/Separated/Never married	47.62%	40.20%	
Education level (%)			0.400
Less than high school	12.25%	9.77%	
High school	38.09%	35.61%	
More than high school	49.66%	54.63%	
Ratio of family income to poverty			0.343
<1.33	30.88%	33.11%	
1.33–3.5	42.65%	36.68%	
≥3.5	26.47%	30.21%	
Smoking (%)			0.003
Yes	55.41%	43.29%	
No	44.59%	56.71%	
Hypertension (%)			<0.001
Yes	63.51%	35.62%	
No	36.49%	64.38%	
Diabetes (%)			<0.001
Yes	28.28%	13.22%	
No	71.72%	86.78%	
TC (mg/dL)	189.42 ± 44.60	191.42 ± 40.95	0.375
HDL (mg/dL)	52.97 ± 18.26	53.10 ± 16.12	0.401
LDL (mg/dL)	107.80 ± 29.87	113.07 ± 35.53	0.273
BMI (kg/cm^2^)	30.21 ± 6.41	29.21 ± 6.89	0.022
Alcohol use status	2.01 ± 2.11	3.74 ± 30.79	<0.001
LAP	82.19 ± 62.67	68.70 ± 63.68	<0.001

Mean ± SD for continuous variables: the *P* value was calculated by the weighted linear regression model.

(%) for categorical variables, the *P* value was calculated by the weighted chi-square test.

Abbreviations: LAP: lipid accumulation product; TC: total cholesterol; HDL: high-density lipoprotein; LDL: low-density lipoprotein; BMI: body mass index.

### Correlation between LAP and colon cancer risk

The univariate and multivariate logit model studies looking at the relationship between LAP levels and colon cancer risk factors are shown in Table 2. For every unit rise in LAP, the colon cancer OR rose by 1.43 (95% CI: 1.18–1.73, ***P*** = 0.0003) in Model 1, which did not account for any confounders. This suggests a strong positive correlation between LAP level and colon cancer risk. Classification based on LAP level quartiles revealed that group Q3 (OR: 2.24, 95% CI: 1.35–3.70, ***P*** = 0.0018) had a significantly higher risk of colon cancer than group Q1, which was the first quartile used as a reference. Furthermore, group Q4 (OR: 2.14, 95% CI: 1.29–3.56, ***P*** = 0.0032) had a substantially greater risk of colon cancer than group Q1. The findings of the accompanying trend analysis and further sensitivity analysis were likewise statistically significant (***P*** for trend 0.0007).

**Table 2:** Associations between LAP (ln transform) and colon cancer.

	OR (95%CI), *P*-value		
	Model 1	Model 2	Model 3
**LAP**	1.43(1.18, 1.73) 0.0002	1.30 (1.04, 1.62) 0.0211	10.56 (2.40, 46.53) 0.0018
**LAP (Quartile)**			
**Q 1**	1.00	1.00	1.00
**Q 2**	1.37 (0.79, 2.37)	0.88 (0.50, 1.53)	3.71 (0.95, 14.45)
	0.2679	0.6417	0.0584
**Q 3**	2.24 (1.35, 3.70)	1.36 (0.82, 2.27)	7.34 (1.25, 42.88)
	0.0018	0.2385	0.0269
**Q 4**	2.14 (1.29, 3.56)	1.44 (0.86, 2.42)	7.68 (0.53, 111.14)
	0.0032	0.1656	0.1349
***P* for trend**	0.0007	0.0497	0.0424

Model 1: No adjustments;

Model 2: Minimally adjusted for Age, Gender, and Race.

Model 3: Fully adjusted for Age, Gender, Race, Marital status, Education level, Ratio of family income to poverty, Smoking, Alcohol use status, Hypertension, Diabetes, TC, HDL, LDL, and BMI.

Abbreviations: LAP: lipid accumulation product; TC: total cholesterol; HDL: high-density lipoprotein; LDL: low-density lipoprotein; BMI: body mass index.

The OR for colon cancer per unit rise in LAP level in Model 2 that was corrected for age, gender, and race was 1.30 (95% CI: 1.04–1.62, ***P*** = 0.0211). Furthermore, when comparing the Q3 and Q4 groups to the Q1 group for reference, the risk of colon cancer was not statistically significant (Q3: OR: 1.36, 95% CI: 0.82–2.27, ***P*** = 0.2385; Q4: OR: 1.97, 95% CI: 1.14–3.42, ***P*** = 0.016). Nevertheless, the outcomes of the additional sensitivity analyses and the accompanying trend analyses continued to be statistically significant (***P*** for trend 0.0497).

Once more factors were taken into account in model 3, including marital status, education level, the ratio of family income to poverty, smoking, alcohol use status, hypertension, diabetes, TC, HDL, LDL, and BMI, the OR for colon cancer increased significantly with each unit increase in LAP level, coming in at 10.56 (95% CI: 2.40–46.53, ***P*** = 0.0018). The Q3 group (OR: 7.34, 95% CI: 1.25–42.88, ***P*** = 0.0269) had a considerably higher risk of colon cancer than the Q1 control group. Additionally, the trend analysis and additional sensitivity analyses produced results that were still statistically significant (***P*** for trend 0.0424).

### Subgroup analysis

**Fig 2:**
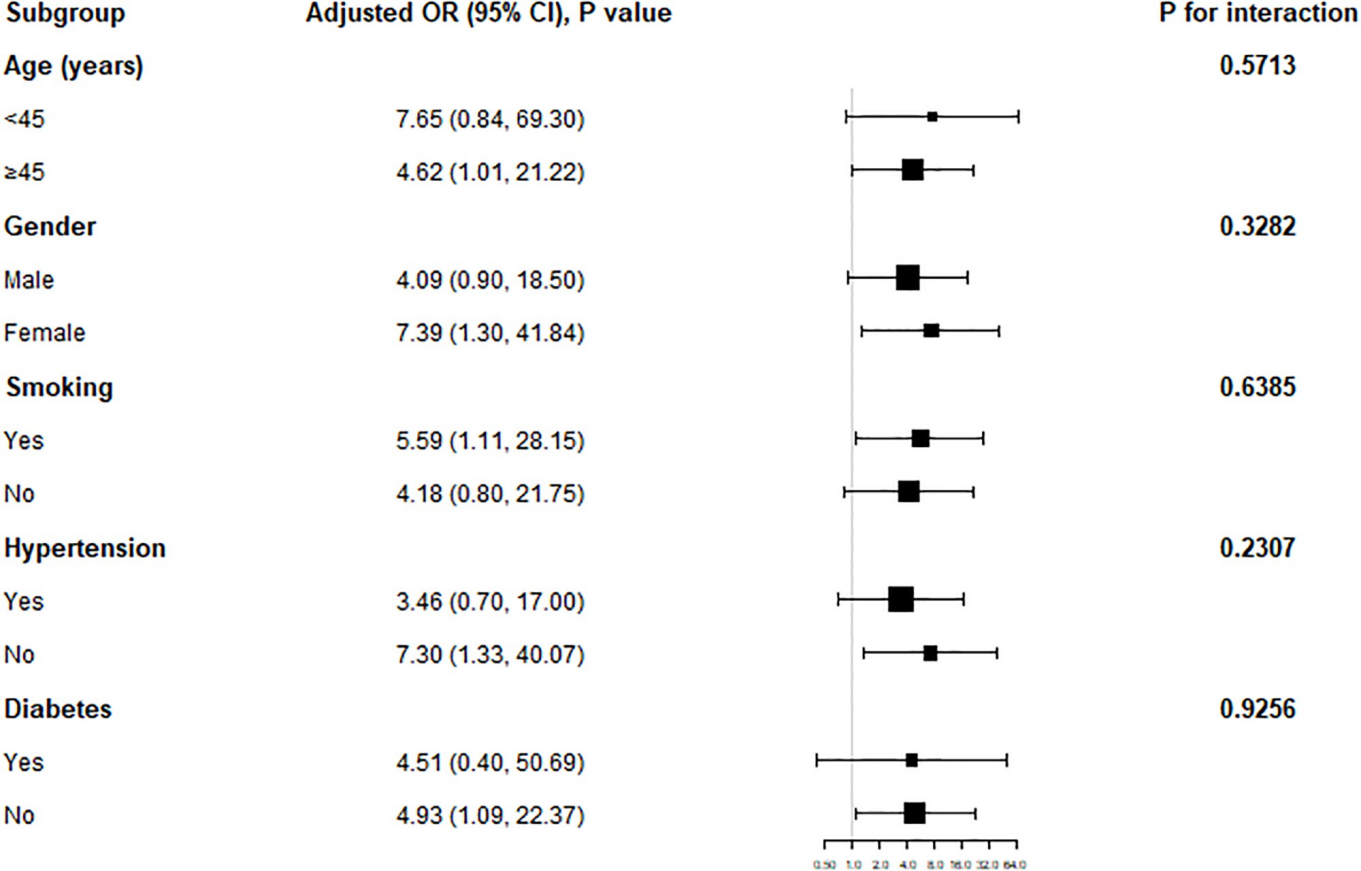
Subgroup analysis.

As illustrated in Fig 2, our study was further analyzed in subgroups according to age, gender, smoking, hypertension, and diabetes and tested for interaction to further validate the correlation between LAP level and colon cancer risk in the general population and to ascertain the specificity in the population. Our findings demonstrated that the risk of colon cancer increased considerably with rising LAP levels in the female population, in individuals aged ≥45 years, in Smoking, in individuals who did not have hypertension, and in individuals who did not have diabetes. there was no significant interaction in the other subgroups (All P for interaction > 0.05).

**Fig 3:**
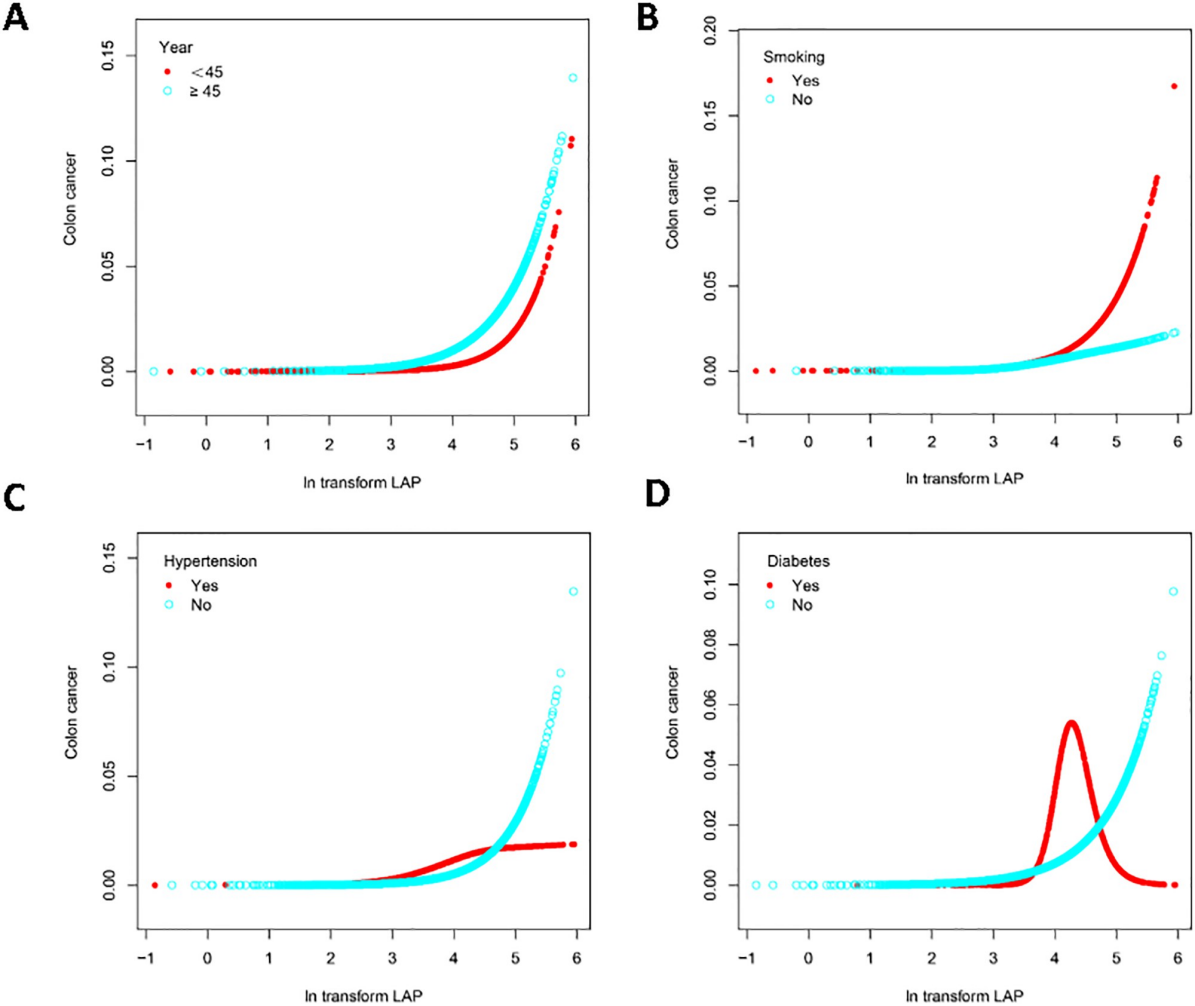
Stratified smoothing curve fitting.

Furthermore, using further stratified smoothing curve fitting, we investigated the nonlinear association between LAP levels and colon cancer risk in several subgroups, as illustrated in Fig 3. We found a significant inverse L-shaped relationship between LAP levels and colon cancer risk in people aged 45 years and older (Fig 3A), as well as an inverse L-shaped relationship in smokers and people without hypertension (Fig 3B and 3C), and to a V-shaped relationship in people with diabetes (Fig 3D).

## Discussion

In this work, we examined the association between LAP levels and colon cancer risk using data from the National Health and Nutrition Examination Survey (NHANES) from 2009 to 2018. According to our research, the risk of colon cancer increases with the amount of LAP present in the organism. After controlling for age, gender, race, marital status, education level, the ratio of family income to poverty, smoking, alcohol use status, hypertension, diabetes, TC, HDL, and BMI, the connection remained significant. Furthermore, we investigated the demographic specificity of the association between LAP levels and colon cancer risk using subgroup analysis. The population under 60 years old, the female population, the population without smoking, the population without hypertension, and the population without having diabetes showed the strongest correlation between LAP levels and colon cancer risk. Furthermore, the nonlinear connection between LAP levels and colon cancer risk in several demographic subgroups was investigated using further stratified smoothed curve fitting. These findings add to our understanding of the connection between colon cancer risk and organismal LAP levels and offer insightful information that will help shape future public health risk assessment techniques.

This is reportedly the first large-scale cross-sectional study that looks into the potential relationship between colon cancer risk and LAP levels. Prior research has linked obesity and related markers to an increased risk of colon cancer [23–26]. A greater BMI was linked to a higher risk of colorectal cancer development in both men and women, according to observational research (OR: 1.27; 95% CI: 1.17, 1.38); when gender is taken into account, both men (OR: 1.29; 95% CI: 1.15, 1.44) and women (OR: 1.25; 95% CI: 1.10, 1.41) have a greater risk of CRC with higher BMI [27]. Waist-to-Hip Index and specifically A Body Shape Index were strongly correlated with colon cancer in both men and women in Mendelian randomization research [28]. Furthermore, LAP is an emerging measure that incorporates the TG indicator for lipid metabolism along with WC levels [29]. Additionally, several studies have demonstrated a link between TG levels and the risk of colon cancer [30, 31]. After multivariate correction, a 6% increase in colon cancer risk was linked to every 1-SD rise in TG levels in a prospective cohort study of the UK biobank [32]. In a Chinese retrospective study, individuals in the Q2, Q3, and Q4 groups of the triglyceride-glucose index (TyG) had a risk of colon tumors that was 1.324, 1.349, and 1.445 occasions greater than those in the Q1 group, respectively. They also had a risk of recurrence that was 2.267 occasions greater in the Q3 and Q4 groups than in the Q1 group, after confounders were taken into account [33]. As an additional illustration, a study found that a higher TyG index was linked to a higher risk of colon cancer [34]. Our results point to the potential need for public health interventions to lower organismal LAP levels, as greater LAP levels may be linked to an increased risk of colon cancer.

The biological processes that underlie these connections are quite intricate. The properties and metabolic activities of fat accumulation in various bodily areas vary comparatively, as adipose tissue is not a uniform solid structure. A meta-analysis of the researched findings revealed that for every 25 cm2 increase in visceral adipose tissue, the probability of colon tumor increased by 13% [35]. The precise processes, nevertheless, that relate the risk of colon cancer to adipose tissue are still up for debate. According to a recent study, visceral adipose tissue positively correlates with markers associated with inflammation and angiogenesis, particularly with circulating levels of vascular endothelial growth factor, which stimulates angiogenesis [36]. Another illustration would be the relatively significant and very active role that adipose tissue plays in the innate immune response. Visceral obesity-associated chronic low-level inflammation all through the body is caused by adipose tissue and immune cells infiltrating into visceral adipose tissue switching from an inflammation-reducing and inhibitory adipogenic phenotype to one that is the pro-inflammatory and pro-adipogenic phenotype [37–39]. This physical inter-conversion offers relatively advantageous circumstances for tumor development. This shows that visceral adipose tissue and LAP levels in the body are significantly correlated with tumor cells and that these relationships may have an effect on how quickly colon cancer patients’ cancers advance. The aforementioned study suggests that adipose tissue from the abdomen or immune cells, which might be among the methods of effect, have a direct or indirect impact on the immunological microenvironment when it comes to the risk of LAP in colon cancer.

The findings of our investigation strength is the use of a sizable, statistically representative information set, which improves how applicable the results are to the adult population in the United States. To reduce the influence of confounders on the outcomes, extensive adjustments for matching possible confounders were also performed. Furthermore, further stratified smoothed curve fitting and subgroup analyses were employed to provide a more versatile depiction of the connection between organismal LAP levels and colon cancer risk.

Our study does have several limitations, though. First, we were unable to establish a causal link between organismal LAP levels and colon cancer risk due to the cross-sectional analysis carried out using the NHANES data. Secondly, memory bias could have been introduced to some extent by using questionnaires for a few of the variables. Then, even though we made the necessary adjustments for several confounders, the rule is still likely that some confounders, which include the hereditary history with relatives, history of abdominal electromagnetic radiation, and inflammatory bowel illness, exist since they were either insufficiently or unappreciated. To further elucidate the causal link, several prospective research, as well as pertinent fundamental studies, must be conducted as follow-up investigations.

## Conclusions

To sum up, our research indicates a favorable association between organismal LAP levels and colon cancer risk. Increased organismal LAP levels might be a colon cancer risk factor. Subsequent longitudinal investigations will be required to validate our results and delve more into the plausible processes behind the observed correlation.

## Abbreviations

BMI: body mass index
Cm: centimeter
CRC: colorectal cancer
HDL: high-density lipoprotein
LAP: lipid accumulation product
LDL: low-density lipoprotein
NHANES: National Health and Nutrition Examination Survey
TC: total cholesterol
TyG: triglyceride-glucose index
